# Plasma Gelation as an Overlooked Determinant of Transfection Efficiency in Protein Corona‐Coated Lipid Nanoparticles: Implications for In Vitro–In Vivo Translation

**DOI:** 10.1002/adhm.71646

**Published:** 2026-08-26

**Authors:** Serena Renzi, Luca Digiacomo, Francesca Giulimondi, Daniela Pozzi, Junbiao Wang, Augusto Amici, Cristina Marchini, Chiara Cassone, Alessandra Zingoni, Fabrizio Marra, Luca Buccini, Francesco Mura, Valentina De Lorenzi, Francesco Cardarelli, Giulio Caracciolo

**Affiliations:** ^1^ Department of Molecular Medicine Sapienza University of Rome Rome Italy; ^2^ Department of Medical Surgical Sciences and Biotechnologies Sapienza University of Rome Latina Italy; ^3^ School of Biosciences and Veterinary Medicine University of Camerino Camerino Italy; ^4^ Department of Astronautics Electrical and Energy Engineering Sapienza University of Rome Rome Italy; ^5^ Research Center on Nanotechnologies applied to Engineering (CNIS) Sapienza University of Rome Rome Italy; ^6^ Department of Basic and Applied Sciences for Engineering (SBAI) Sapienza University of Rome Rome Italy; ^7^ Laboratorio NEST Scuola Normale Superiore Pisa Italy

**Keywords:** bio‐nano interactions, in vitro transfection, lipid nanoparticles, protein corona

## Abstract

A common observation in drug delivery studies is that preincubation of nanoparticles (NPs) with high concentrations of human plasma (HP) markedly reduces in vitro transfection efficiency (TE). This effect is commonly attributed to the formation of a protein corona (PC), which is assumed to impair cellular uptake or intracellular trafficking of gene delivery systems such as lipid nanoparticles (LNPs). However, the evidence presented here suggests an alternative mechanism underlying this phenomenon. To reproduce conditions in which LNPs encounter an excess of circulating proteins, NPs were incubated in HP prior to exposure to cells in standard calcium‐containing culture medium. Under these conditions, plasma undergoes gelation, leading to the formation of a clot‐like network. A combination of complementary approaches, including dynamic light scattering (DLS), fluorescence‐activated cell sorting (FACS), confocal fluorescence microscopy, raster image correlation spectroscopy (RICS), and functional assays evaluating TE and cell viability, demonstrates that this gel‐like matrix restricts LNP diffusion in the extracellular environment. In contrast, coronated LNPs that reach the intracellular space display comparable trafficking behavior, indicating that the PC does not compromise intracellular processing. These findings highlight the importance of extracellular factors when evaluating the impact of the PC and extrapolating in vitro results to in vivo settings.

## Graphical abstract

Upon contact with plasma, lipid nanoparticles (LNPs) rapidly acquire a protein corona (PC) that redefines their biological identity. In calcium‐containing environments, these coronated particles trigger the formation of a gel‐like plasma network, profoundly altering their transport properties and reducing their effective availability. This extracellular barrier restricts access to the cell surface, limits membrane engagement, and ultimately compromises intracellular delivery and transfection efficiency (TE).

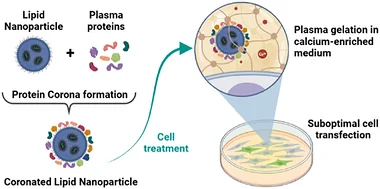

## 1 Introduction

Lipid nanoparticles (LNPs) have emerged as a transformative platform in gene therapy [1, 2]. Their clinical success, most notably demonstrated by the regulatory approval of *Onpattro* [3], followed by the rapid development and deployment of mRNA vaccines during the COVID‐19 pandemic [4, 5], has demonstrated the potential of lipid‐based systems for safe and effective nucleic acid delivery [1, 6]. LNPs are designed to encapsulate and protect nucleic acid cargo within their lipid structures, facilitating delivery to target cells while minimizing nuclease‐mediated degradation and immune detection [7]. Due to ease of manufacture, reduced immune responses, multi‐dosing capabilities, and flexibility of design, LNPs are considered the leading nonviral delivery systems [2]. Their physicochemical properties, including composition, proportion of the components, particle size, surface characteristics, and functionalization, can be finely tuned and tailored to optimize cellular uptake and endosomal escape, which represent critical steps for efficient transfection [8, 9].

Yet, an additional key factor should be considered: when nanoparticles (NPs) are introduced into biological media (e.g., blood, serum, or plasma), they rapidly interact with surrounding biomolecules, resulting in the adsorption of a biomolecular layer on their surface [10, 11]. This layer is primarily composed of proteins and is therefore commonly referred to as the protein corona (PC), as originally termed by Dawson and coworkers [12, 13]. Although the adsorption kinetics and molecular composition of the corona depend on numerous interdependent factors, including the type and physicochemical properties of NPs, the biological medium, and environmental shaping factors [14, 15], the formation of a PC is considered an inevitable process for any system exposed to a biological medium [16]. In gene delivery, nanocarriers acquire a PC that alters their surface properties and impacts their biological behavior by affecting biodistribution, immune recognition, cellular uptake, and exocytosis [17], and ultimately their therapeutic efficacy [18]. To ensure physiologically relevant conditions and generate reliable predictions of functional NP performance, such effects should be studied under preincubation settings with excess plasma (typically >50%), which better mimic the in vivo protein‐rich environment [19]. In this context, it is documented in the current literature that the PC formed at physiological concentrations typically reduces the transfection efficiency (TE) of different gene‐delivery vectors, including polymeric carriers [20], graphene hybrids [21], and LNPs [22].

The decrease in TE observed with PC formed at high protein concentrations has been primarily attributed to impaired endosomal escape and enhanced lysosomal accumulation [20, 21, 22]. In this context, the cell tends to direct the NPs toward recycling endosomes, whereas the associated PC is preferentially trafficked to multivesicular bodies [23].

However, while the inhibitory effects of the PC on NP performance are well documented, additional factors may be implicated. For instance, beyond the direct effects of the PC, changes in the physicochemical properties of the extracellular environment, such as increased medium viscosity, may also contribute. Interactions between plasma proteins and calcium ions present in widely used cell culture media such as Dulbecco's Minimal Essential Medium (DMEM) or Roswell Park Memorial Institute (RPMI) medium have been shown to induce plasma gelation [24]. This process results in gel‐ or clot‐like structures that alter rheological properties of the medium [25, 26]. Calcium ions are key regulators of the coagulation cascade, essential for activating several factors [27], and can directly stimulate platelet activation and clot formation [28]. While citrate‐anticoagulated plasma remains fluid in vitro, adding calcium restores coagulability and promotes clotting [29].

It is worth noting that plasma gelation is not inherently detrimental: in specific applications, it has been intentionally harnessed to generate biocompatible scaffolds for cell culture. In particular, freshly prepared autologous plasma clots formed by calcium‐induced coagulation have been used as a carrier matrix for mesenchymal stromal cells, allowing high intra‐clot cell viability and proliferation when optimized calcium concentrations are applied [25]. However, under standard in vitro transfection settings, such gelation may instead act as a physical and biochemical barrier, impairing NP diffusion and cell access.

Building on this rationale, we systematically investigated how PC formation together with calcium‐induced plasma gelation affects the TE of five multicomponent LNPs encapsulating plasmid DNA (pDNA), referred to as F1 to F5. To include multiple design parameters, our formulations differed in lipid composition and molar ratio, preparation technique, and surface functionalization. A preliminary characterization revealed the physical‐chemical properties of the systems both in buffer and in human plasma (HP). Then, we performed in vitro TE experiments in calcium‐containing versus calcium‐free media, aiming to disentangle the effects of the PC from those of possible plasma gelation. Our findings indicate that the reduced TE of LNPs exposed to high concentrations of HP is not solely attributable to PC formation but is significantly influenced by calcium‐induced plasma gelation. This gel‐like barrier hinders NP access to cells, thereby limiting functional delivery. These results underscore the importance of accounting for extracellular physical constraints, such as medium gelation, when designing in vitro experiments aimed at faithfully recapitulating in vivo conditions.

## 2 Results and Discussion

**FIGURE 1:**
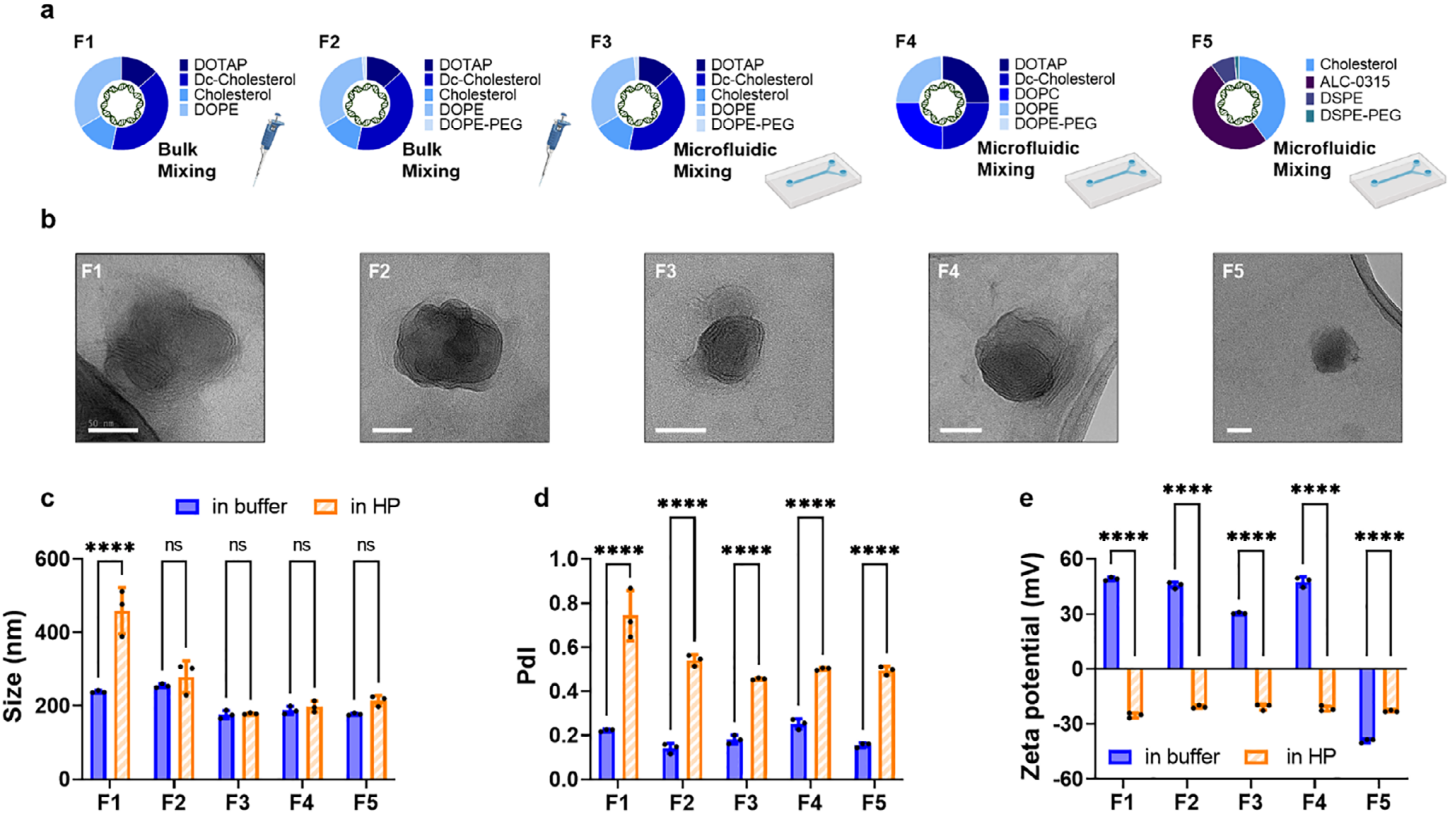
Physicochemical characterization of bare and coronated LNPs. (a) Lipid compositions and schematic of the lipid‐DNA assembly procedure used to prepare formulations F1–F5. (b) Representative TEM images of F1–F5 (scale bar: 50 nm). (c) Average size (determined by main peak position), (d) polydispersity index (PdI), and (e) zeta potential for F1–F5. Data are presented as mean ± SD (*N* = 3 technical replicates). Statistical analysis was performed by two‐way ANOVA followed by Šidák's post hoc test. Statistical significance is indicated as follows: n.s. = not significant; *p < 0.05, **p < 0.01, ***p < 0.001, and ****p < 0.0001.

In this study, we prepared, characterized, and tested five multicomponent LNPs encapsulating pDNA. The selected formulations differed in lipid composition (i.e., lipid species and relative molar ratios), surface functionalization (i.e., unPEGylated vs. PEGylated systems), and preparation method (bulk mixing vs. microfluidic mixing). These choices were designed to represent a range of commonly used strategies in LNP‐mediated gene delivery, thereby enabling a broader interpretation of the results. Comprehensive formulation parameters for F1–F5 are listed in Table 1, and a schematic overview is shown in Figure 1a. Representative transmission electron microscopy (TEM) images of all the formulations are reported in Figure 1b. The images reveal discrete NPs with a predominantly spherical morphology and a characteristic internal organization, consistent with the multilayered structure commonly observed in multicomponent LNPs. Further TEM images for F1–F5 are provided as Supporting Information (Figure S1).

**TABLE 1:** Lipid composition, functionalization, and mixing procedure for formulations F1–F5.

		F1	F2	F3	F4	F5
**Lipid amount (molar ratio)**	DOTAP	0.132	0.132	0.132	0.250	0.000
	DC‐cholesterol	0.400	0.400	0.400	0.250	0.000
	ALC‐0315	0.000	0.000	0.000	0.000	0.500
	Cholesterol	0.133	0.133	0.133	0.000	0.400
	DOPC	0.000	0.000	0.000	0.250	0.000
	DSPC	0.000	0.000	0.000	0.000	0.085
	DOPE	0.335	0.320	0.320	0.245	0.000
	DOPE‐PEG2k	0.000	0.015	0.015	0.005	0.000
	DSPE‐PEG2k	0.000	0.000	0.000	0.000	0.015
**Functionalization**		Plain	PEG	PEG	PEG	PEG
**Mixing with pDNA**		Bulk	Bulk	Microfluidic	Microfluidic	Microfluidic

After preparation, all systems were exposed to commercially available HP (1:1 v/v) for 1 h at 37°C. These incubation conditions follow established protocols designed to reproduce the physiological milieu encountered by NPs in vivo [30, 31, 32]. Incubation of LNPs with HP under these conditions leads to the spontaneous formation of LNP‐PC complexes (hereafter indicated as “coronated LNPs”). A comprehensive physicochemical characterization of both bare and coronated LNPs is reported in Figure S2 and summarized in Figure 1b–d. Globally, bare formulations displayed unimodal size distributions centered between 160 and 250 nm, whereas upon HP exposure, a main peak accompanied by two distinct smaller‐sized populations was clearly distinguishable (Figure S1). These secondary peaks, centered within 10–20 nm and 30–80 nm, respectively, are attributed to the excess of proteins in solution, as documented in previous studies on the coronated NPs [32, 33]. In contrast, the main peak represents the average size of coronated LNPs, which were consistently larger than the bare counterparts. For the sake of clarity, the general trends of the measured physical‐chemical properties for F1–F5 are reported in Figure 1b–d, which also offer a closer examination of formulation‐dependent behaviors. For instance, the greatest size increase upon incubation with HP was observed for the unPEGylated formulation F1, whereas its PEGylated counterpart F2 and the other PEGylated systems F3, F4 exhibited only slight changes (Figure 1b). This pronounced size increase in F1 can be attributed both to extensive protein adsorption from HP, leading to the formation of a thick PC, and to particle aggregation [34]. In fact, unPEGylated systems are more prone to aggregation due to reduced colloidal stability and increased tendency for protein adsorption [35]. In contrast, PEG chains create a steric barrier, effectively reducing protein binding and limiting both size changes and particle clustering [36]. Incidentally, the formulation prepared by microfluidic mixing underwent the smallest size increase after exposure to HP, and these trends were consistent with those observed for the polydispersity index (PdI) (Figure 1c). Specifically, all the bare formulations initially exhibited low PdI values after preparation (i.e., 0.12–0.25) but showed increased PdI upon exposure to HP, reaching 0.74 for F1, 0.54 for F2, 0.46 for F3, and 0.50 for F4 and F5. Finally, zeta potential measurements (Figure 1d) showed that, regardless of the formulation, the initial surface charge of the pristine LNPs (cationic for F1–F4, anionic for F5) reached a common value upon incubation with HP. This trend is a well‐established effect, commonly referred to as “*zeta potential normalization*,”, and is due to the adsorption of negatively charged plasma proteins onto the particle, and results in the convergence of initially diverse surface charges toward a similar negative value under physiological conditions [37, 38].

Overall, our initial characterization shed light on the physicochemical properties of LNPs in both buffer and HP. The pristine F1–F5 formulations had small size and low PdI, making them suitable for nucleic acid delivery. Following exposure to HP, they rapidly acquired a PC, yielding larger and negatively charged coronated LNPs with increased heterogeneity. These coronated particles represent the biologically relevant entities that operate in vivo and were therefore used as model systems to evaluate delivery performance under physiologically meaningful conditions.

**FIGURE 2:**
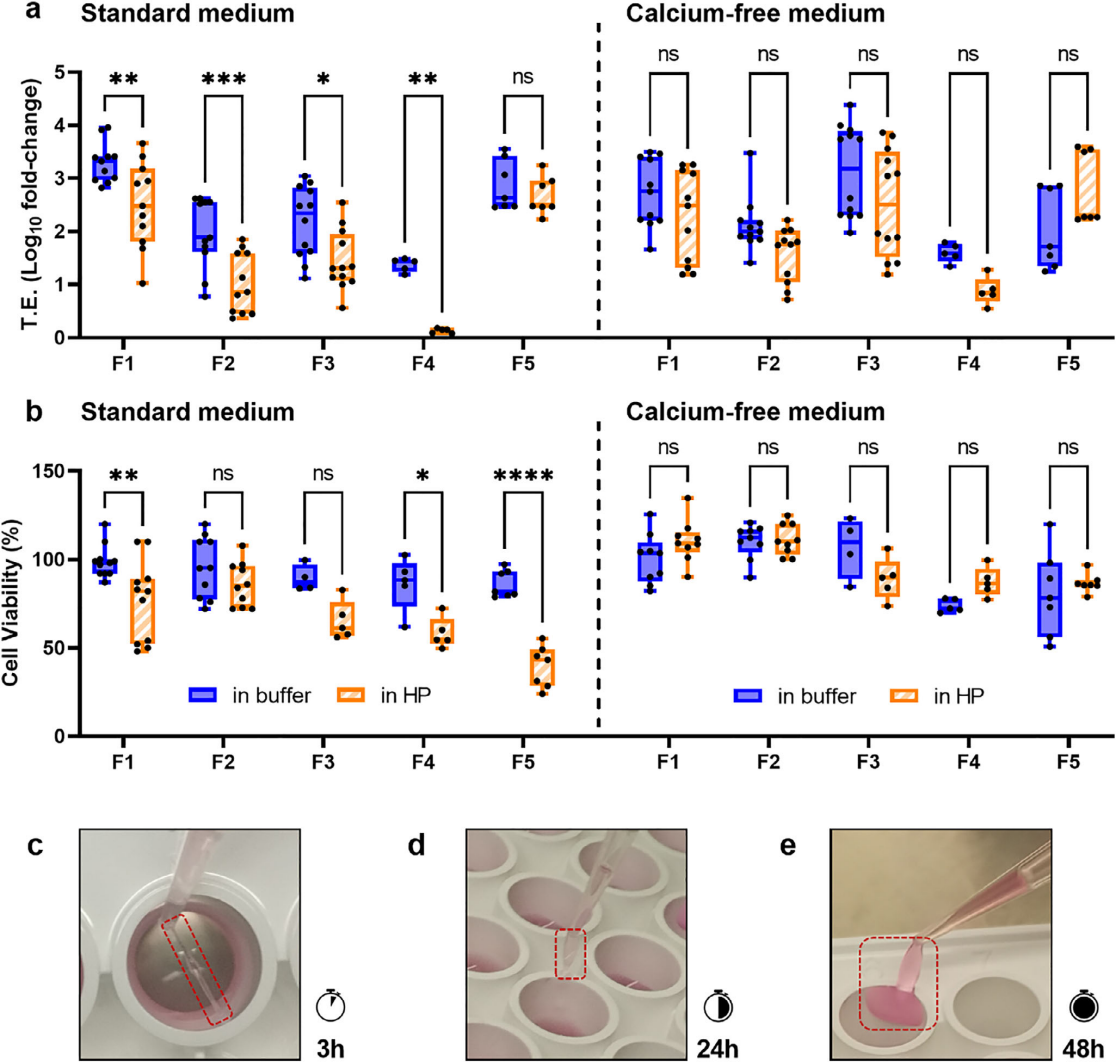
Effect of calcium‐dependent gelation of culture medium on LNP transfection and viability. (a) TE of formulations F1–F5 and (b) corresponding cell viability in both standard culture medium and calcium‐free medium. Representative images of the macroscopic appearance of NP‐plasma mixtures in culture medium containing calcium at different time points: (c) 3, (d) 24, and (e) 48. Data are presented as box plots, minimum‐to‐maximum whiskers; *N* ≥ 5 biological replicates. Statistical analysis was performed by two‐way ANOVA followed by Šidák's post hoc test. Statistical significance is indicated as follows: n.s. = not significant; *p < 0.05, **p < 0.01, ***p < 0.001, and ****p < 0.0001.

To this end, we treated HEK‐293 cells with F1–F3, F5 and AsPC‐1 with F4. HEK‐293 cells are widely used in nanomedicine research owing to their robust growth and well‐characterized behavior, making them an ideal model for a nonspecialized human cell line [39]. On the other hand, hard‐to‐transfect pancreatic cancer cells, AsPC‐1, better mimic a pathophysiological target environment and offer complementary insights into the transfection behavior of our systems in disease‐relevant contexts. Notably, these cells represent a valuable model for evaluating local, intraoperative delivery strategies in pancreatic ductal adenocarcinoma (PDAC), where systemic treatment remains challenging due to the poor vascularization and limited drug accessibility of this tumor. TE of F1–F5 is reported in Figure 2a, along with the corresponding cell viability (Figure 2b). TE is expressed as logarithmic fold change with respect to control (i.e., not treated) cells. Overall, in standard cell culture medium, unPEGylated F1 exhibited the largest TE in buffer, followed by its PEGylated counterparts F2 and F3, whereas F4 exhibited the lowest TE (Figure 2a). This observation is consistent with existing literature, which documents a decrease in TE for PEGylated systems. This effect is commonly attributed to the so‐called “PEG dilemma” [40], wherein PEGylation enhances colloidal stability and extends systemic circulation yet concurrently hampers cellular uptake and intracellular release of the therapeutic cargo. Although further considerations about formulation‐dependent behaviors can be made and related to features and composition of the systems, a more general trend can be easily recognized, that is, the significant reduction of their TE upon exposure to HP. Indeed, in standard medium, coronated cationic LNPs exhibited statistically significant lower TE than their bare counterparts (i.e., those suspended in buffer), except for F5 that is the only formulation containing ionizable lipids and exhibiting an anionic surface charge. This characteristic likely mitigates the impact of PC formation, which is primarily driven by electrostatic interactions between proteins and nanosystems and is typically enhanced by a net positive surface charge.

Contextually, cell viability in standard medium following treatment with bare formulations remained high, averaging between 86% and 98%. However, when LNPs were incubated with HP, cell viability was reduced in a formulation‐dependent manner, ranging from 44% to 86% (Figure 2b). Interestingly, we conducted parallel experiments using calcium‐free medium, and our experimental evidence clearly indicates that under this condition, the reduction in TE upon exposure to HP was markedly attenuated. Additionally, in calcium‐free medium, cell viability remained high for both bare and coronated formulations.

Notably, when administered to cells, the formulations form a visible clot or gel‐like aggregate only upon dispersion in standard culture medium containing calcium. This observation suggests a macroscopic phase transition, likely triggered by calcium‐induced plasma gelation. Representative images of coronated LNPs added to standard cell culture medium at different time points (3, 24, and 48 h) are provided in Figure 2c–e and reveal the progressive formation of a visible gel‐like structure. At 3 h, the medium remained largely liquid, with early signs of initial aggregation visible along a filamentous pattern. By 24 h, a gel‐like structure was more evident, and at 48 h, a prominent semi‐solid clot was observed, clearly retained by the pipette tip during aspiration. Beyond visual inspection, scanning electron microscopy (SEM) and rheological analyses were performed to provide complementary structural and functional characterization of the gelation phenomenon. SEM images of the isolated gelled component (Figure S3) revealed a heterogeneous, interconnected dried matrix composed of elongated, fibrillar‐like features and irregular interstitial spaces. Although sample dehydration may alter the native architecture of the soft gel, these observations support the presence of an organized network within the gelled material. Rheological measurements were then used to determine whether this network translated into measurable changes in the flow properties of the hydrated system. Three conditions were analyzed: (i) standard cell‐culture medium, (ii) calcium‐free medium containing coronated F1, which remained liquid by visual inspection, and (iii) the F1–HP mixture in standard calcium‐containing medium, in which gelation occurred. As shown in Figure S3, the gelled sample exhibited markedly increased viscosity and pronounced shear‐thinning behavior, whereas the control medium and the calcium‐free formulation showed predominantly liquid‐like, near‐Newtonian responses. Together, the morphological and rheological findings provide complementary evidence that calcium promotes the formation of an interconnected soft‐gel network, resulting in a substantial alteration of the viscosity and flow behavior of the mixture.

**FIGURE 3:**
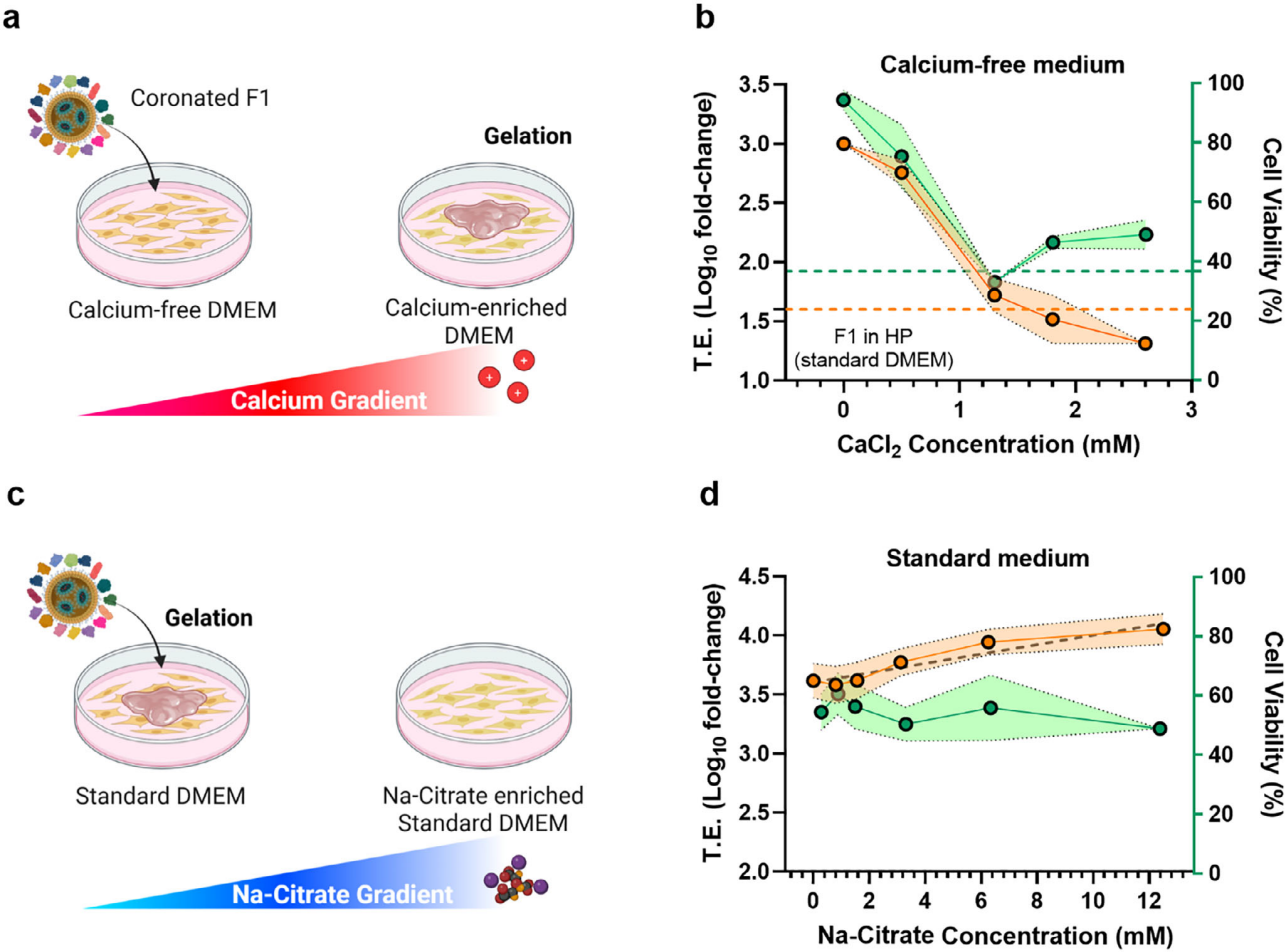
Effects of cell culture media enrichment with calcium or sodium citrate. (a) HEK‐293 cells were treated with coronated F1 in calcium‐free DMEM supplemented with increasing concentrations of calcium chloride (CaCl_2_, 0–2.6 mM per well) for 3 h. Afterward, the medium was replaced with fresh standard DMEM. (b) TE (orange dots, left *y*‐axis) and cell viability (green dots, right *y*‐axis) were measured after 48 h. The long‐dashed line indicates the TE of coronated F1 in standard medium (used as reference), while the short‐dashed line indicates cell viability after the same treatment. (c) HEK‐293 cells were treated with coronated F1 in standard DMEM supplemented with increasing concentrations of sodium citrate (0–12.5 mM per well) for 3 h. (d) TE (left *y*‐axis) and cell viability (right *y*‐axis) were measured after 48 h. The dashed black line indicates the increasing linear trend of TE as a function of Na‐citrate concentration. Shaded areas indicate the standard deviation across *N* ≥ 4 independent experiments, reflecting the variability of transfection efficiency and cell viability measurements.

Since all five formulations displayed the same qualitative response to HP exposure under standard and calcium‐free conditions, F1 was selected as a representative model for the subsequent mechanistic investigations owing to its higher TE and the more pronounced differences observed between the experimental conditions. In this regard, a further indication about the role of calcium as responsible for plasma gelation in culture media was obtained by investigating the effect of calcium chloride (CaCl_2_) supplementation to calcium‐free medium. We evaluated the TE and cytotoxicity of the representative coronated formulation F1 after treatment in media enriched with calcium at four concentrations of Ca^2^^+^: (i) 0.5, (ii) 1.2, (iii) 1.8, and (iv) 2.6 mM (Figure 3a). The lowest concentration is below physiological levels; the second corresponds to the free calcium concentration in plasma; the third matches that of standard DMEM; and the highest simulates the total (free plus chelated) calcium concentration in plasma. As shown in Figure 3b, increasing calcium concentration led to a progressive reduction in TE (orange) and cell viability (green), consistent with the onset of plasma gelation, which became macroscopically visible at concentrations approaching or exceeding those of plasma (≥1.2 mM). Interestingly, the TE and cell viability measured in calcium‐free medium supplemented with 1.2 and 1.8 mM CaCl_2_ closely resembled those observed for coronated F1 in standard medium, as indicated by the colored dashed lines in the graph. In this experiment, we applied the same experimental protocol used for cells treated in calcium‐free medium—specifically, the removal of medium after 3 h of treatment, which exacerbated the reduction in cell viability observed. This effect is likely due to gel formation, which caused detachment and loss of cells upon medium removal.

Having established that the reduction in TE is closely associated with calcium‐dependent gel formation and the resulting changes in the rheological properties of the medium, we next asked whether calcium might also directly influence transfection by altering the composition of the PC. To this end, we compared the composition of the corona preformed on F1 following HP exposure after dispersing the coronated particles in standard DMEM, calcium‐free DMEM, or calcium‐free DMEM supplemented with physiological CaCl_2_ (Figure S4). Although a comprehensive comparison and discussion of these results is provided in the Supporting Information, including Figure S4 and the accompanying explanatory text, the key observation relevant here is that the overall PC profiles formed in calcium‐free medium and calcium‐free medium supplemented with CaCl_2_ were similar, with comparable total protein content and only minor differences in the intensity of selected bands. These findings suggest that calcium alone had a limited impact on the global composition of the corona.

Finally, to correlate the effect of gel formation with TE, we analyzed coronated F1 in standard medium while varying the amount of sodium citrate, with the aim of preventing or reversing gel formation at a fixed calcium concentration of media (1.8 mM). As represented in Figure 3c, we used increasing amounts of sodium citrate, from a minimum concentration of 0.8 mM to a maximum of 12.5 mM (approaching citrate concentration in commercial plasma). Interestingly, increasing the amount of sodium citrate restored the system's ability to reach and transfect cells, as gel formation was impaired or reversed (Figure 3d). To determine whether the recovery of TE could be attributed to a direct enhancing effect of sodium citrate, rather than to its ability to prevent gel formation, bare F1 was tested at the highest citrate concentration. As shown in Figure S5, sodium citrate did not increase TE; instead, it caused an overall reduction in the TE of bare F1 in both standard and calcium‐free media, possibly due to mild cytotoxicity, maybe due to slight toxicity for cells. Furthermore, additional characterization studies indicated that sodium citrate did not affect the particle size and only marginally influenced PdI (Figure S6), thus not impacting on the colloidal stability of the formulation. In addition, further SDS‐PAGE analysis (Figure S7) indicated that sodium citrate induced only secondary changes in the overall PC composition over the investigated concentration range, suggesting that the recovery of TE was predominantly associated with calcium chelation and the inhibition of plasma gelation rather than with substantial citrate‐induced alterations of the PC.

To determine whether the TE reduction induced by gelation was restricted to commercial plasma containing sodium citrate as an anticoagulant, we also tested plasma obtained from healthy human donors anticoagulated with a distinct calcium‐chelating agent, that is, K_2_EDTA. In addition, to assess whether differences in the protein composition of the plasma could influence this effect, we evaluated TE and cell viability in HEK‐293 cells for each specific donor. Results are shown in Figure S8 and indicate that, regardless of the donor, all plasma samples induced gelation in standard medium, thereby impairing the efficiency of the tested gene delivery systems. Interestingly, no such effects were observed when treatments were performed in calcium‐free medium. Although it is well established that the PC is personalized and that individual physiological variability and health status influence both PC composition and NP‐cell interactions [41, 42, 43], under our experimental conditions, designed to closely mimic in vivo NP‐plasma interactions (i.e., using an excess of plasma and avoiding centrifugation steps to isolate the hard corona), the observed phenomenon appeared generalized. Specifically, all plasma samples led to gel formation that reduced TE and cell viability, effects that could otherwise be misattributed to PC composition. These findings underline the importance of minimizing artefacts associated with plasma‐induced gel formation to avoid potential misinterpretations in studies investigating the impact of personalized PCs on NP‐cell interactions. Interestingly, we performed parallel experiments using heparin as an anticoagulant. However, heparin itself exerted a non‐negligible and deleterious effect on TE (Figure S9), making it unsuitable for studying the relationship between PC formation and gene delivery system performance. This is consistent with previous reports showing that heparin can inhibit cationic carrier‐mediated transfection without affecting cell viability [44]. While our study primarily addresses the phenomenological consequences of plasma gelation on LNP TE, NP‐plasma interactions at the molecular interface may influence local gel formation. LNPs are pre‐incubated in plasma prior to Ca^2^^+^ reconstitution, leading to rapid PC formation and a shift to a net anionic surface charge, as confirmed by zeta potential measurements. The inhibitory effect of heparin (Figure S9) indicates the involvement of plasma coagulation components rather than a purely physicochemical Ca^2^^+^‐anticoagulant equilibrium. Our data are therefore consistent with a coagulation‐driven gel network incorporating corona‐coated LNPs, although a contributory role of the corona‐mediated molecular interface in shaping local gel organization cannot be excluded. Altogether, our results suggest that calcium‐induced plasma gelation compromises NP bioavailability by promoting their physical separation from the cell monolayer, thus hindering cellular uptake, leading to reduced TE. In addition, the formation of a gel‐like matrix likely restricts the diffusion of essential nutrients and oxygen to the adherent cells, leading to a decline in cell viability. This phenomenon is consistent with observations in hydrogel systems, where limited diffusion results in reduced metabolic activity and increased cell death [45]. To investigate this hypothesis, we employed biophysical methods alongside fluorescence labeling and confocal microscopy to track LNP mobility in both extracellular and intracellular environments under gelation and non‐gelation conditions, aiming to elucidate the relationship between diffusion limitations, TE, and cell viability.

**FIGURE 4:**
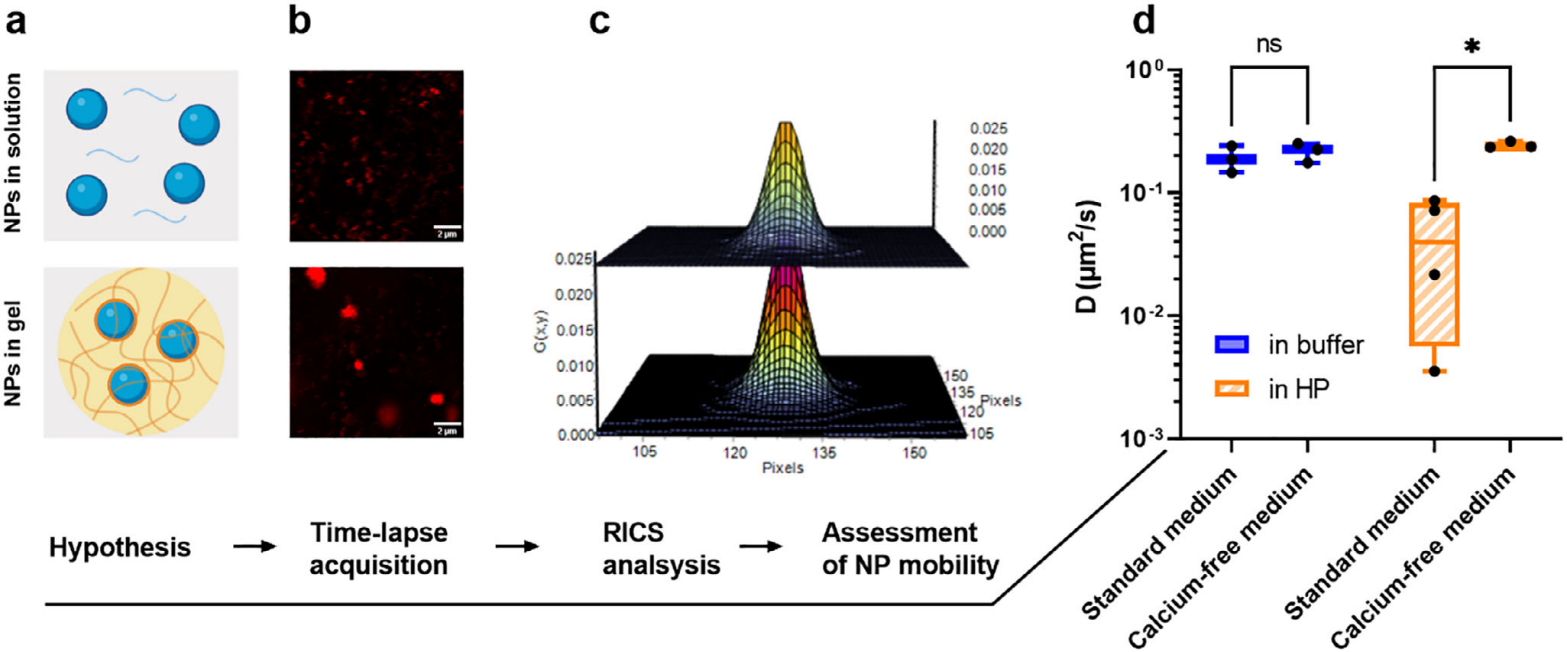
(a) Cartoon depicting the reduction of particle mobility in gel. (b) Maximum intensity projection of representative time‐lapse acquisition. (c) Exemplary RICS correlation function (bottom) together with fit (top). (d) Diffusion coefficients measured by RICS analysis. HEK‐293 cells were treated for 3 h with F1 formulations pre‐incubated in buffer or exposed to HP, either in standard medium or calcium‐free medium. Diffusion measurements were performed in the extracellular medium. Data are presented as box plots, minimum‐to‐maximum whiskers, *N* ≥ 3. Statistical analysis was performed by two‐way ANOVA followed by Šidák's post hoc test. Statistical significance is indicated as follows: n.s. = not significant; *p < 0.05, **p < 0.01, ***p < 0.001, and ****p < 0.0001. Panel a was created with BioRender.com.

A direct observation of reduced particle mobility within the gel was obtained using raster image correlation spectroscopy (RICS), a consolidated technique for measuring particle dynamics in solutions and living cells with standard confocal microscopes [46, 47, 48]. As a representative model, F1 was fluorescently labeled, exposed to HP, and incubated in either standard medium or calcium‐free medium. Based on our previous observations, in calcium‐free medium NPs should move rapidly in solution, whereas in standard medium NP mobility is expected to be limited by particle sequestration within the gel matrix, as schematically illustrated in Figure 4a.

To verify this interpretation, time series of confocal fluorescence images (Figure 4b) were acquired and processed by RICS to compute the corresponding correlation functions (Figure 4c) and finally quantify the particle diffusion coefficients. Results are shown in Figure 4d and clearly indicate that LNPs not exposed to HP (i.e., in buffer) incubated in either standard medium or calcium‐free medium exhibited the same diffusion coefficients, approximately 0.20  ±  0.04 µm^2^/s. In contrast, when LNPs were preincubated with HP, a marked difference emerged: their diffusion was significantly lower in standard medium, where calcium triggers plasma gelation, compared to calcium‐free medium, where gelation is prevented. Specifically, the diffusion coefficient in the gelled medium dropped to about one‐seventh of that in the calcium‐free condition. This sharp reduction in mobility suggests that the gel acts as a dense physical network that traps or restricts movement of LNPs, creating a substantial barrier to cell interaction and internalization.

**FIGURE 5:**
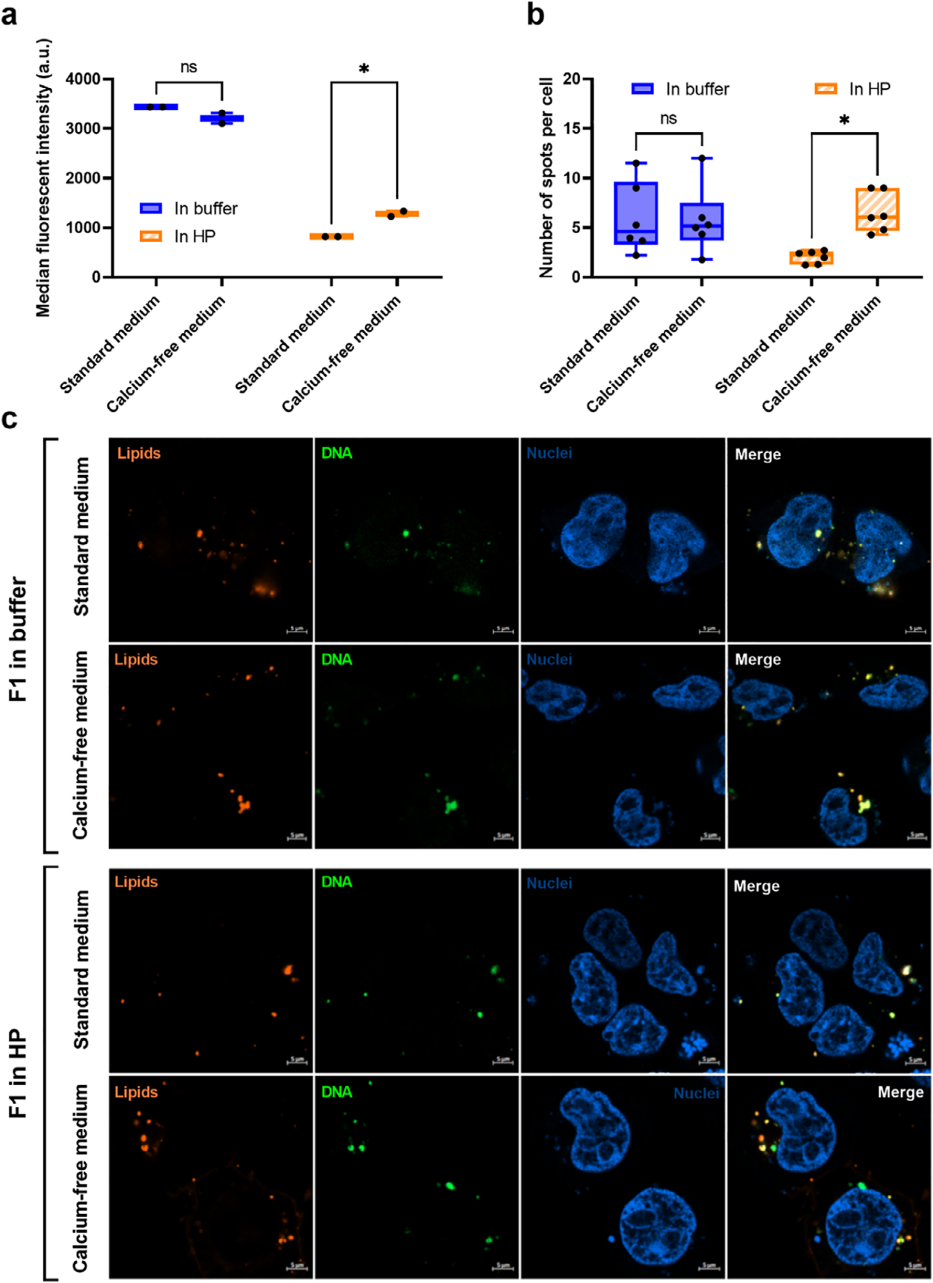
(a) Median fluorescence intensity by flow cytometry experiments revealing the uptake of F1 by HEK‐293 cells, in buffer or upon exposure to HP, both in standard and calcium‐free medium. (b) Number of fluorescent spots per cell corresponding to F1 under the explored conditions, obtained by fluorescent confocal microscopy. (c) Representative confocal images of the investigated samples. Statistical analysis was performed using two‐way ANOVA, followed by Šidák's post hoc test (*N* = 2 for FACS analysis, *N* ≥ 5 for confocal microscopy).

As a next step, we investigated whether the presence of the extracellular gel influences LNP cellular uptake and intracellular behavior. To this end, we performed fluorescence‐activated cell sorting (FACS) cytometry experiments on HEK‐293 cells following a 3‐h treatment with fluorescently labeled F1 in buffer or after exposure to HP, both in standard and calcium‐free media. As shown in Figure 5a, F1 in HP was internalized to a lesser extent than the bare counterpart, particularly in standard medium, where the decrease in median fluorescence intensity was more pronounced. Importantly, this trend is consistent with the formation of an extracellular gel matrix that hinders LNP uptake by the cells. Interestingly, the FACS data (all the distributions are provided in Figure S10) were corroborated by fluorescence confocal microscopy experiments (Figure 5b). Specifically, both the lipids and the DNA components of F1 were fluorescently labeled, along with the cell nuclei. Two main observations can be drawn from the corresponding confocal images (Figure 5c): (i) a reduced number of particles per nucleus was detected when F1 was exposed to HP and administered in standard medium, and (ii) a strong correlation between the lipid and DNA signals was observed (colocalization coefficients are reported in Figure S11.

In summary, FACS analysis demonstrated that plasma gelation (i.e., in the case of HP‐exposed LNPs in standard medium) significantly reduces cellular uptake, and this finding was independently confirmed by single‐cell confocal fluorescence microscopy. The reduced uptake, reflected in a lower number of particles per cell, likely represents the primary cause of the decreased TE observed under plasma gelation conditions.

**FIGURE 6:**
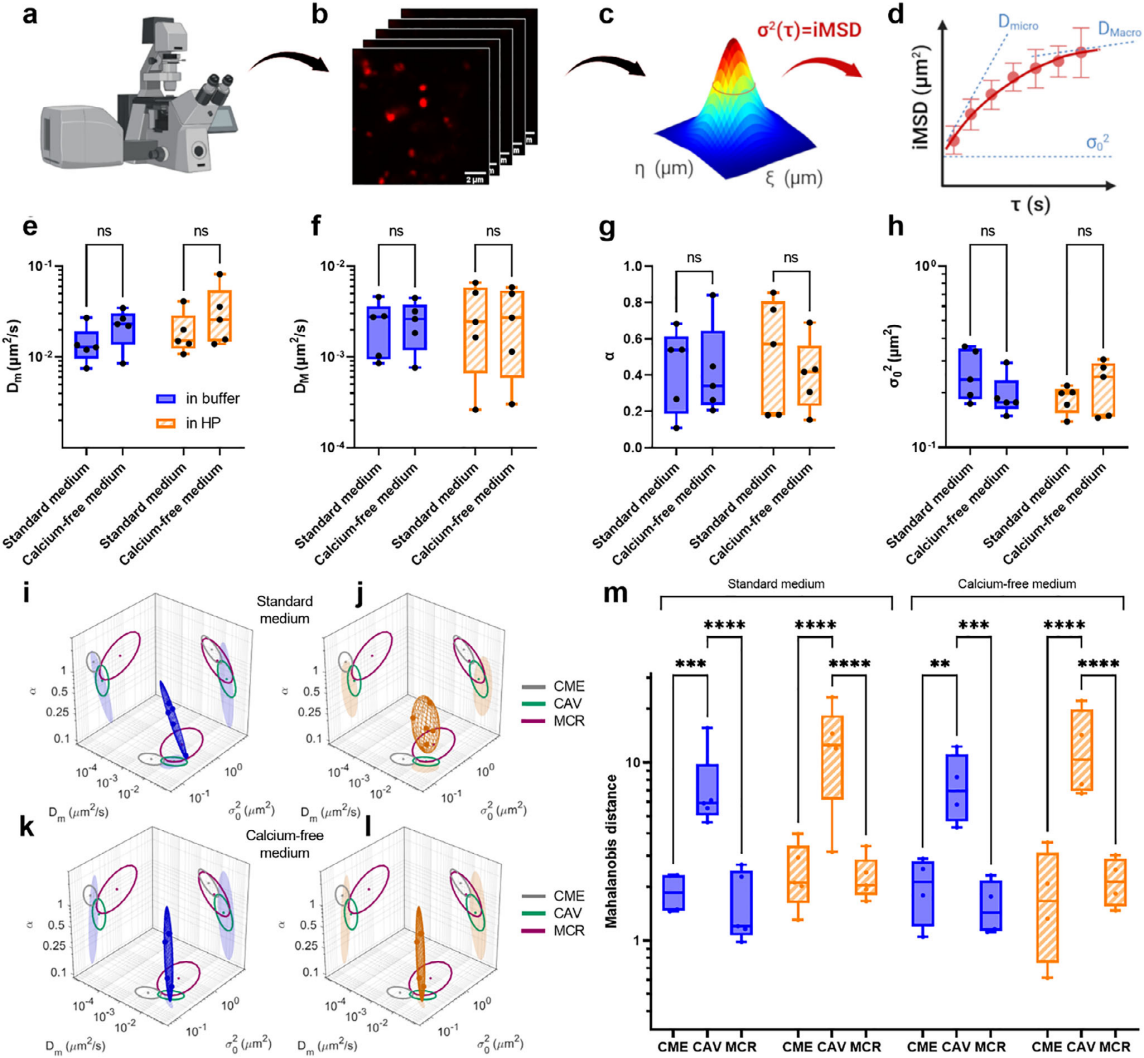
Schematic representation of the image‐derived mean square displacement (iMSD) approach. (a) Confocal fluorescence time‐lapse acquisition yields (b) an image time series, from which the (c) spatial‐temporal correlation function is computed. (d) The resulting variance *σ* ^2^(*τ*) is fitted versus the temporal lag variable. For all the investigated samples, Diffusion coefficients at (e) short and (f) long time scales were determined from the iMSD slope; deviations from pure Brownian diffusion were quantified by (g) the alpha value (power law), and (h) the average spot size *σ*_0_^2^ was estimated from the *y*‐intercept. The example shown refers to the F1 formulation administered to HEK‐293 cells (*N* = 5). (i–l) 3D scatterplot of the obtained dynamic parameters, compared to those corresponding to CME, CAV, and MCR (whose data are reported with permission from Refs [49, 50]). (m) Mahalanobis distances from CME, CAV, and MCR reference distributions. Statistical analysis was performed using two‐way ANOVA, followed by Šidák's post hoc test. Panel a was created with BioRender.com.

We next focused on characterizing F1 dynamics at the cellular level to elucidate the underlying internalization mechanism. To this end, we employed the image‐derived mean square displacement (iMSD) approach (details are given in the Experimental section), a spatial‐temporal correlation technique that extracts dynamic information from time‐lapse confocal image sequences by quantifying how the fluorescence signal of moving particles decorrelates over time and space (Figure 6a–d). This method allows for a label‐free, ensemble measurement of intracellular mobility directly from fluorescence intensity fluctuations within live cells, providing a reliable readout of nanoscale dynamics without requiring particle tracking. As shown in Figure 6, iMSD analysis performed on HEK‐293 cells treated with F1 revealed that both buffer‐ and HP‐treated LNPs display comparable dynamic behavior. Specifically, the analyzed samples exhibited similar subdiffusive dynamics, with comparable diffusion coefficients at both short and long time scales, as well as similar spot sizes (Figure 6e–h). Notably, the measured dynamic parameters were compared with those obtained for representative endocytic vesicles associated with distinct internalization pathways, namely clathrin‐mediated endocytosis (CME), caveolae‐mediated endocytosis (CAV), and macropinocytosis (MCR), as illustrated in the 3D scatter plot (Figure 6i–l). To quantitatively assess the dissimilarity between the sample distributions and the reference vesicle populations, the Mahalanobis distance was calculated. The resulting values (Figure 6m) indicate that, under all tested conditions, F1 is not internalized via CAV (i.e., exhibited a large distance from the CAV reference distribution), but rather through CME and MCR to a comparable extent.

Collectively, the data presented in Figures 4, 5, 6 support the conclusion that plasma gelation impairs LNP bioavailability primarily at the extracellular level, by limiting the fraction of LNPs that can successfully interact with and enter cells, without affecting their intracellular behavior and final fate. Evidence regarding LNP final fate is provided in Figure S10, which reports colocalization analyses with lysosomal markers and shows no significant differences of LNP lysosomal degradation under the explored conditions. It should be noted that the mechanistic investigations presented here were performed in HEK‐293 cells, a well‐established model for nonviral gene delivery studies. However, the same qualitative response was observed in AsPC‐1 cells treated with F4, supporting the broader relevance of the identified mechanism. Further validation in additional cell types will nevertheless be valuable.

Importantly, macroscopic gelation should not be interpreted as a process independent of PC dynamics. Rather, it likely reflects a calcium‐driven reorganization of the entire system, including plasma proteins, medium components, and proteins associated with the LNP surface. In this context, calcium availability may simultaneously promote gel formation and induce structural or compositional rearrangements of the PC, which cannot be disentangled under these conditions. To contextualize the observed effects and critically assess the adopted experimental design, we next examine the rationale and implications of exposing LNPs to a protein‐rich environment prior to cellular administration. This “pre‐coating” strategy is a widely adopted in vitro approach to approximate the biological identity that NPs acquire in vivo and involves their pre‐incubation with biological fluids to promote the formation of a PC prior to cellular exposure [51]. In our experiments, LNPs were pre‐incubated in 50% HP and subsequently added to cells without any intermediate purification step, thereby retaining an excess of free proteins in the suspension. Of course, this protocol introduces the possibility that excess proteins may contribute to the formation of macroscopic gel or clot‐like aggregates in the culture medium, and it could be argued that isolating the pre‐coated LNPs from unbound proteins (e.g., by size exclusion chromatography [52, 53]) could indeed prevent such aggregation. However, our study followed a different rationale: although many investigations aim to explore cellular interactions of NPs bearing a stable PC, it is important to acknowledge that studying these interactions in a protein‐depleted medium may fail to capture the complexity of the in vivo environment. Moreover, if the separation step is introduced specifically to avoid plasma‐induced gelation, it is important to consider that even corona‐coated NPs, once purified, may release loosely bound proteins when resuspended in a protein‐free culture medium. This release is driven by thermodynamic equilibrium, as proteins with low binding affinity tend to dissociate in the absence of competing free proteins, potentially reintroducing conditions that favor aggregation or gel‐like phase transitions. Indeed, we observed gelation phenomena even at lower plasma concentrations, and sub‐visible gelation may still occur even in the absence of macroscopically detectable aggregates. These subtle phase transitions can create physical barriers that affect both TE and cell viability. Therefore, it is essential to employ complementary analytical techniques, such as turbidity and viscosity measurements, to detect early‐stage gelation events that are not visually evident but can still compromise the interpretation of functional assays. Finally, it should be noted that, although plasma gelation emerged as a major determinant of LNP transport in the present study, it represents only one of several physiological variables that contribute to the in vivo performance of nanomedicines. Additional factors, including the dynamic evolution of the PC across different biological compartments [19, 54, 55], blood flow and associated shear forces [33, 56], endothelial permeability, and interactions with cells of the mononuclear phagocyte system [57], are also expected to influence NP biodistribution, cellular uptake, and therapeutic efficacy. Future in vitro platforms integrating these physiological cues will therefore be instrumental in further narrowing the gap between conventional cell culture assays and in vivo outcomes.

## 3 Conclusion

In this work, we assessed the in vitro TE of five LNPs encapsulating pDNA, both in their pristine and coronated form. Transfection assays conducted in standard culture medium revealed a pronounced reduction in TE for all coronated formulations, which correlated with visible clot formation. This gel‐like aggregation led to physical entrapment of the LNPs, limiting their diffusion and availability for interaction with cells, and contributed to a reduction in cell viability. Notably, this phenomenon was absent when calcium‐free medium was used, under which the TE of the coronated LNPs was restored to levels comparable to the pristine systems.

This observation also identifies a practical strategy to overcome calcium‐dependent gelation during in vitro transfection experiments, whereby the use of calcium‐free transfection conditions enables the evaluation of the intrinsic transfection performance of coronated LNPs while minimizing aggregation‐related artifacts.

**FIGURE 7:**
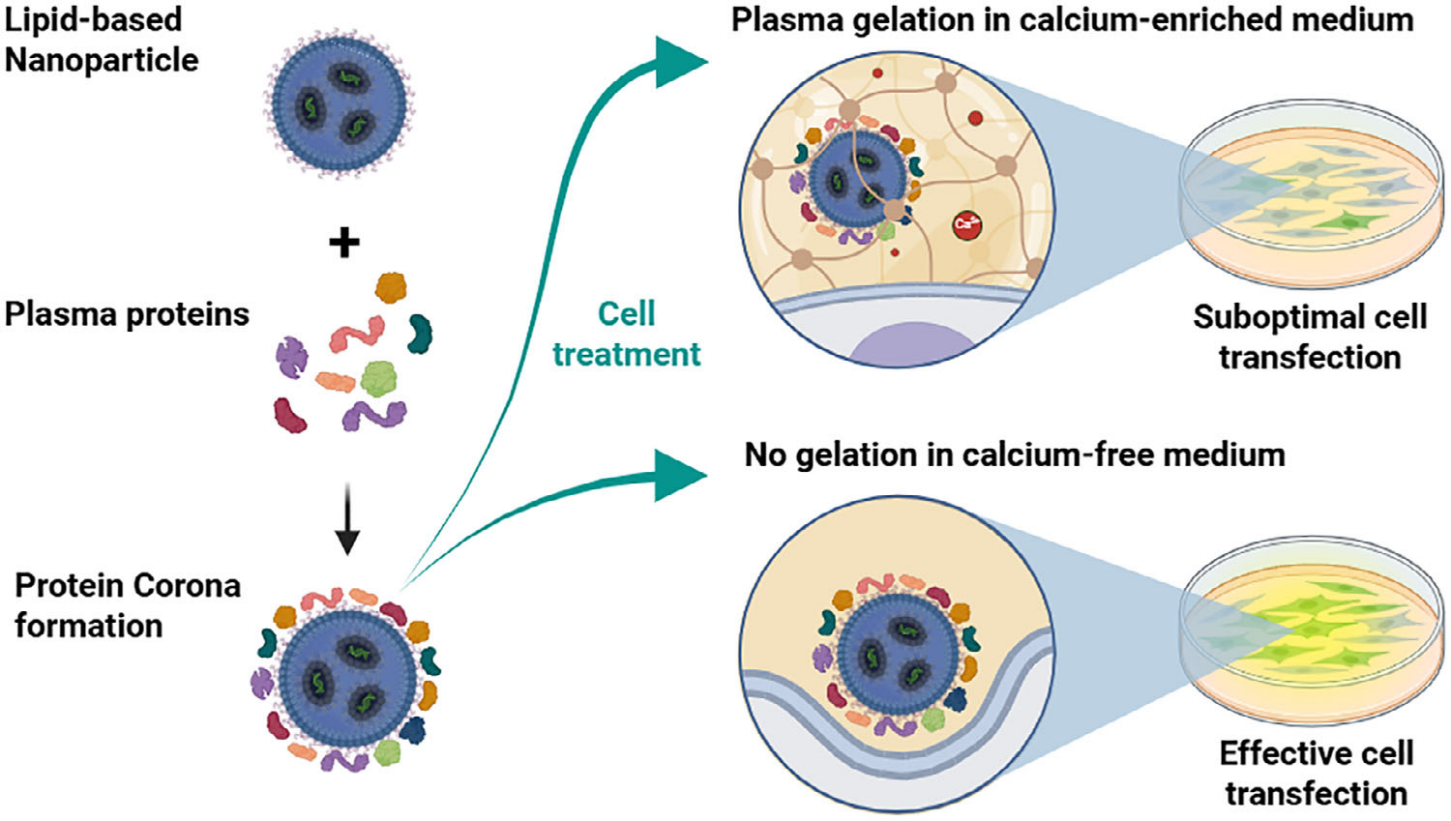
Schematic representation of the impact of plasma gelation on the cellular transfection of LNPs. Upon incubation with plasma proteins, LNPs acquire a PC. In calcium‐containing culture medium, plasma gelation leads to the formation of a dense extracellular matrix, limiting NP diffusion and cellular access, resulting in suboptimal transfection. In contrast, in calcium‐free medium, no gelation occurs, allowing efficient NP‐cell interactions and effective transfection. Created with BioRender.com.

Our findings, schematically summarized in Figure 7, reveal plasma‐induced gelation as a previously overlooked contributing factor to reduced gene TE. This phenomenon, likely associated with the presence of free calcium ions, should be considered when interpreting in vitro transfection outcomes. Notably, this gelation should be regarded as a macroscopic manifestation of calcium‐dependent changes in protein–protein and protein–NP interactions, potentially involving concomitant rearrangements of the PC, rather than as an isolated or purely physical barrier. Neglecting this aspect risks misattributing the relative contributions of PC effects and calcium‐dependent aggregation phenomena, potentially leading to an overestimation of the intrinsic performance of gene delivery systems. Therefore, it is important to identify early‐stage gelation events that are not visibly detectable but may nonetheless compromise the interpretation of functional assay results. By addressing this variable, researchers can achieve more reliable in vitro‐to‐in vivo translation and accelerate the development of delivery platforms with greater predictive and therapeutic power. In summary, our study does not merely provide methodological insights; rather, it might have a significant translational value. Indeed, the plasma gelation we observed in vitro might reflect the activation of blood clotting by cationic LNPs in vivo, as recently reported [58].

## 4 Experimental Section

### 4.1 Preparation of Lipid‐Based NPs

1,2‐Dioleoyl‐3‐trimethylammonium‐propane (DOTAP), 3ß‐[N‐(N',N'‐dimethylaminoethane)‐carbamoyl]cholesterol hydrochloride (DC‐Cholesterol∙HCL), 1,2‐dioleoyl‐sn‐glycero‐3‐phosphocholine (DOPC), 1,2‐dioleoyl‐sn‐glycero‐3‐phosphoethanolamine‐*N‐*polysarcosine‐25 (ammonium salt) (DOPE), 1,2‐distearoyl‐sn‐glycero‐3‐phosphocholine (DSPC), 1,2‐distearoyl‐*sn*‐glycero‐3‐phosphoethanolamine‐*N*‐[amino(polyethylene glycol)‐2000] (DSPE‐PEG(2000)), and 1,2‐dioleoyl‐sn‐glycero‐3‐phosphoethanolamine‐*N*‐[amino(polyethylene glycol)‐2000] (ammonium salt) (DOPE‐PEG 2000) were purchased from Avanti research (Birmingham, AL, USA). Ionizable lipid 4‐hydroxybutyl(azanediyl)bis(hexane‐6,1‐diyl)bis(2‐hexyldecanoate) (ALC‐0315) was purchased from MedChemExpress (Monmouth Junction, NJ, USA), while Cholesterol was obtained from Sigma‐Aldrich (Merck, Darmstadt, Germany). Formulations F1 and F2 were obtained by bulk mixing of liposomes and plasmid DNA (pDNA). Liposomes were prepared by dissolving proper amounts of lipids in chloroform (as indicated in Table 1), then subjected to rotary evaporation for 1 h to form a thin lipid film. The lipid film was hydrated with Milli‐Q water to a final lipid concentration of 1 mg/mL for 4 h. The resulting liposome suspension was extruded 20 times through polycarbonate membranes using a Mini‐Extruder (Avanti Polar Lipids), then incubated 10:1 vol/vol with pDNA (concentration 1 mg/mL) for 20 min at room temperature. Formulations F3, F4, and F5 were prepared by weighing lipids individually, dissolving them in ethanol until a concentration of 12.5 mM, and mixing them at the molar ratios reported in Table 1. The pDNA was dissolved in acetate buffer 25 mM, (pH 4) until a concentration of 233 µg/mL and combined with the ethanol‐lipid phase by microfluidic mixing at a flow rate ratio of 1:3 (ethanol:aqueous) using the NanoAssemblr Ignite system (Precision Nanosystems, Vancouver, Canada), with a total flow rate of 2 mL/min. To reduce residual ethanol (final concentration 25% v/v), the resulting LNP suspension was dialyzed against 1× phosphate‐buffered saline (PBS) for 19 h at 4°C.

### 4.2 Chemical‐Physical Characterization

After a 100‐fold dilution in ultrapure water, the hydrodynamic diameter and PdI of each formulation were measured by dynamic light scattering (DLS) using a Zetasizer Ultra Red (Malvern Panalytical, Malvern, UK). Zeta potential was determined by electrophoretic light scattering (ELS) using the same instrument.

### 4.3 Transmission Electron Microscopy

Samples for imaging were diluted to 1 mg/mL using ultrapure water and deposited onto formvar/carbon‐coated copper grids (Ted Pella 01801), glow‐discharged for 120 s (Pelco easiGlow). Grids were stained with GR‐1000 UA‐Zero EM stain (Agar Scientific), washed in ultrapure water, and dried in air. Samples were examined using a JEOL JEM‐F200 transmission electron microscope at 200 kV, with images captured by a GATAN Rio 16 CMOS camera.

### 4.4 Scanning Electron Microscopy

F1+HP sample embedded in standard medium for 3 h was deposited and left to air‐dry. Afterward, to prevent charging, it was metallized with a 10 nm Cr layer using a Quorum Technologies Q150T ES sputter coater. The analysis was performed using a Zeiss Auriga field‐emission scanning electron microscope (FE‐SEM), operating at 1.5 kV.

### 4.5 SDS‐PAGE Experiments

NPs were incubated with an equal volume of HP (50% v/v final concentration) for 1 h at 37°C to allow PC formation. Subsequently, the NP suspensions were diluted in the appropriate media (standard media, calcium‐free media, or media supplemented with CaCl_2_ or sodium citrate) and further incubated for 3 h under the same conditions used for cell treatments. The resulting NP suspensions were centrifuged at 14 000 rpm for 15 min at 4°C. The supernatant was discarded, and the pellet was resuspended in H_2_O and centrifuged again. This washing step was repeated three times. The final pellet was resuspended in 1× Laemmli buffer supplemented with 10% NuPAGE reducing agent, loaded onto an SDS‐PAGE gel (4%–12% gradient), and electrophoretically separated. After 1 h of electrophoresis, the gel was collected, and protein bands were visualized using a ChemiDoc imaging system (Bio‐Rad). Gel analysis was performed using custom MATLAB scripts, as previously described [7, 59].

### 4.6 In Vitro Experiments

HEK‐293 and AsPC‐1 cell lines (purchased from ATCC, American Type Culture Collection) were cultured in appropriate media, DMEM for HEK‐293 and RPMI for AsPC‐1, supplemented with 10% fetal bovine serum (FBS) and 1% penicillin/streptomycin. For transfection experiments and cell viability experiments, cells were seeded in 96‐well plates at a density of 1 × 10^5^ cells/well and incubated overnight at 37°C with 5% CO_2_. Each well was treated with a fixed amount of encapsulated pDNA (0.25 µg) using 5 µL/well of formulations at a concentration of 0.05 mg/mL. For the PC study, lipid‐based formulations were incubated with human plasma (HP; Sigma‐Aldrich, Merck KGaA, Darmstadt, Germany) at 37°C for 1 h in a 1:1 v/v ratio (5 µL HP per well). As reported by the provider company, HP prepared from pooled human blood contained 4% trisodium citrate as an anticoagulant. For the preparation of heparinized plasma, commercial HP was transferred into a BD Vacutainer LH tube (68 U.I.) and subsequently collected and diluted with non‐heparinized plasma to obtain the desired final heparin concentrations (2–16 U/mL).

For treatment with formulations, the culture media were replaced with serum‐free DMEM or calcium‐free serum‐free DMEM. After 3 h, an equal volume of fresh DMEM supplemented with 20% FBS was added to cells treated in standard DMEM. In contrast, the calcium‐free DMEM containing the treatment was completely replaced with standard DMEM supplemented with 10% FBS. This difference in procedure was necessary to address the cell detachment caused by gelation of plasma (observed in standard DMEM) during media removal. In calcium‐free DMEM, medium replacement was performed to prevent gelation upon addition of calcium‐containing DMEM, while ensuring cell morphology was maintained. After 48 h, cell viability was assessed using the CellTiter‐Fluor Cell Viability Assay (Promega, Madison, WI, USA), following the manufacturer's instructions. Subsequently, the medium was removed, and the cells were lysed using Passive Lysis Buffer (Promega). Half of the resulting cell lysate was used for protein quantification using the Micro BCA Protein Assay Kit (Thermo Fisher Scientific, Waltham, MA, USA), while the remaining lysate was used to measure luciferase expression using the Luciferase Assay System (Promega). Luminescence was quantified with the GloMax Discover Microplate Reader (Promega), and relative light units (RLU) were normalized to the total protein content.

### 4.7 Preparation of Calcium Chloride and Sodium Citrate‐Enriched Media

Calcium chloride (CaCl_2_) and trisodium citrate dihydrate were purchased from Sigma‐Aldrich. A 1 M CaCl_2_ stock solution was prepared in distilled water, filtered through a 0.22 µm membrane, and subsequently diluted in calcium‐free DMEM to obtain final concentrations of 0.5, 1.2, 1.8, and 2.6 mM Ca^2^^+^. Trisodium citrate dihydrate was dissolved in distilled water at 4% (w/v) to yield a 136 mM stock solution, which was sterilized by autoclaving. The citrate solution was added to standard culture medium to obtain final concentrations of 0.8, 1.56, 3.12, 6.25, and 12.5 mM. The resulting calcium‐ and citrate‐enriched media were used for cell treatments with F1 preincubated in plasma (coronated F1).

### 4.8 Rheological Measurements

Rheological measurements were performed at the Interdepartmental Research Center on Nanotechnology Applied to Engineering at La Sapienza University of Rome, using a rotational rheometer (Anton Paar MCR302 system) with a 40 mm cone‐plate configuration with an 80 µm gap. The tests were conducted at a controlled temperature of 37°C using a Peltier system.

### 4.9 Flow Cytometry

To investigate cellular uptake in the HEK‐293 cell line, the F1 formulation was prepared by incorporating Texas Red at a molar ratio of 5/1000 (fluorescent lipid/total lipid, mol/mol). HEK‐293 cells were seeded in 24‐well plates at a density of 5 × 10^4^ cells/well and incubated overnight at 37°C with 5% CO_2_. Each well was treated with 1 µg of pDNA. For treatment with formulations, the culture media were replaced with serum‐free DMEM or calcium‐free serum‐free DMEM. After 3 h, cells were washed with cold PBS and then subsequently analyzed using a FACS Canto instrument from BD Biosciences in San Jose, CA. The cells were gated based on forward versus side scatter to exclude debris, and their specific emission was then assessed. Data analysis was carried out using the software FlowJo_V10.

### 4.10 Raster Image Correlation Spectroscopy

RICS measurements were performed using an Olympus FVMPE‐RS microscope equipped with a two‐photon Ti:sapphire laser (MaiTai HP, SpectraPhysics). Image stacks were acquired as 200‐frame sequences with a pixel dwell time of 2 µs. Each image comprised 256 × 256 pixels, with a pixel size of 50 nm. Texas Red was excited at 800 nm, and fluorophore emission was collected in the 575–645 nm range using a 30× planApo silicon immersion objective (NA  =  1.0). RICS analysis was performed using the simFCS software package (Laboratory for Fluorescence Dynamics, University of California, Irvine; www.lfd.uci.edu), which includes a moving average filter to subtract the contribution of immobile molecules prior to correlation analysis. The software implements the original algorithms described for standard raster scan RICS [46, 47, 48]. Analysis was conducted under the assumption of a 3D diffusion model and yielded diffusion coefficients (D, µm^2^/s) of the labeled LNPs.

### 4.11 Fluorescence Confocal Imaging

Static multichannel confocal imaging was performed on HEK‐293 cells treated with F1 in buffer or after exposure to HP, both in standard and calcium‐free media. Cells were seeded on glass‐bottom 8‐well imaging chambers (Ibidi µ‐Slide 8 Well) and allowed to adhere prior to treatment. For fluorescence imaging, F1 lipids were labeled with Texas Red‐PE, while the encapsulated DNA was labeled with fluorescein. Cell nuclei were stained with Hoechst dye, and lysosomes were labeled using LysoTracker Deep Red according to the manufacturer's instructions. After treatment, cells were washed to remove excess probes and imaged under the appropriate spectral settings to avoid channel cross‐talk. Images were acquired using a Zeiss LSM 980 confocal microscope equipped with a 63× oil‐immersion objective (NA 1.4). Sequential acquisition was performed for each fluorophore to minimize spectral overlap.

### 4.12 Image‐Derived Mean Square Displacement

iMSD measurements were performed using a Zeiss LSM 800 confocal microscope equipped with a 63×, 1.4 N.A. oil immersion objective. HEK‐293 cells were seeded on glass‐bottom dishes (Willco wells # HBST‐3522) 24 h before the experiment. On the day of confocal acquisitions, cells were incubated with the lipid formulations for 3 h at 37°C. Confocal time‐lapse acquisitions (256 × 256 pixels, 50 nm pixel size, 410 ms scan time) were taken with Texas Red excited at 561 nm (HeNe laser), and emissions were collected between 570 and 630 nm. iMSD analysis was performed with custom scripts, publicly available along with all the necessary codes, a manual, and data for a representative example [7]. The spatiotemporal correlation function of the detected intensity was computed, and its Gaussian variance *σ* ^2^ was plotted to obtain the iMSD curve as a function of the lag time, as detailed in previous works [49, 50, 60, 61]. Then, *σ* ^2^(*τ*) was fitted for further analysis. In detail, *σ* ^2^(*τ*) was first fitted to the following power‐law function:

$$\sigma^{2}\;\left(\tau \right)=\sigma_{0}^{2}+\kappa \tau^{\alpha }$$

to determine the *α* value, which is associated with subdiffusive (i.e., *α* < 1), superdiffusive (i.e., *α* > 1) motion, or Brownian diffusion (i.e., *α* = 1). *σ*_0_^2^ represents the curve's intercept and is related to the average size of the fluorescent‐labeled particles and the waist of the point spread function. Finally, to quantify the intracellular dynamics of the particles, the iMSD curve was fitted to [62]

$$\sigma^{2}\;\left(\tau \right)=\sigma_{0}^{2}+4D_{M}\tau +\frac{L^{3}}{3}\left(1-e^{-\frac{\tau }{\tau_{c}}}\right)$$

where *L* is the linear size of the confinement area, *τ*_c_ quantifies how fast confinement occurs, and *D*_M_ is the particle diffusivity on a large time scale. The short‐term diffusivity *D*_m_ is measured by the slope of *σ* ^2^ for *τ* → 0 and reads *D*_m_ = *D*_M_ + *L* ^2^/(12*τ*_c_).

### 4.13 Statistical Analysis

Statistical analysis was performed using GraphPad Prism Version 9.0.0 for each of the conditions explored, the sample size was *n* = 3 technical replicates for the physicochemical characterization, *n* ≥ 4 biological replicates for in vitro experiments (i.e., transfection experiments and cell viability assays), *n* = 3 technical replicates of *n* = 5 biological replicates for HP samples obtained from human donors, *n* ≥ 3 technical replicates for RICS analysis, and *n* = 5 biological replicates for iMSD analysis. ANOVA tests followed by appropriate multiple comparison tests were performed, and the corresponding details are reported in the figure captions along with the statistical significance, which is indicated as follows: n.s. = not significant; *p < 0.05, **p < 0.01, ***p < 0.001, and ****p < 0.0001.

## Author Contributions

**S.R**.: investigation, validation, visualization, and writing – draft and editing. **L.D**.: data curation, formal analysis, investigation, software, validation, visualization, and writing – draft and editing. **F.G**.: investigation and validation. **D.P**.: conceptualization, project administration, and resources. **J.W**.: investigation. **A.A**.: conceptualization, supervision, and writing – review and editing. **C.M**.: conceptualization, methodology, supervision, and writing – review and editing. **C.C**.: investigation. **A.Z**.: conceptualization and supervision. **F.M**.: investigation. **L.B**.: investigation and methodology. **F.Mu**.: investigation and methodology. **V.D.L**.: investigation and validation. **F.C**.: conceptualization, funding acquisition, methodology, supervision, and writing – review and editing. **G.C**.: conceptualization, funding acquisition, methodology, project administration, resources, supervision, and writing – review and editing.

## Funding

The authors acknowledge funding from the European Union – NextGenerationEU through the Italian Ministry of University and Research under PNRR – M4C2‐I1.3 Project PE_00000019 “HEAL ITALIA” to Giulio Caracciolo, CUP: B53C22004000006 and project ECS00000017 “Ecosistema dell” Innovazione’ Tuscany Health Ecosystem (THE, PNRR, Spoke 4: Nanotechnologies for diagnosis and therapy) to Francesco Cardarelli. The research leading to these results has also received funding from the AIRC Foundation for Cancer Research in Italy (ID. 32500 to G.C.). The AIRC Foundation is also acknowledged for supporting S.R. through an AIRC Fellowship for Italy Post‐Doc grant (Project ID 32938‐2025).

## Ethics Statement

Informed and written consent in accordance with the Declaration of Helsinki was obtained from all healthy blood donors, and approval was obtained from the Ethics Committee of the Sapienza University of Rome (Protocol number 639/16 RIF /CF4179).

## Conflicts of Interest

The authors declare no conflicts of interest.

## Use of Generative AI and AI‐Assisted Technologies in the Writing Process

During the preparation of this manuscript, the authors used ChatGPT (OpenAI) exclusively to improve the grammar, clarity, and readability of the manuscript text. The tool was not used for the generation or interpretation of scientific content, data analysis, figure preparation, or drawing scientific conclusions. All AI‐assisted text was carefully reviewed and edited by the authors, who take full responsibility for the content of the manuscript.

## Supporting information




**Supporting File**: adhm71646‐sup‐0001‐SuppMat.docx.

## Data Availability

The data that support the findings of this study are openly available in Zenodo at https://doi.org/10.5281/zenodo.18763832, reference number 18763832.
