# Supplementary material for: Plasma Gelation as an Overlooked Determinant of Transfection Efficiency in Protein Corona‐Coated Lipid Nanoparticles: Implications for In Vitro–In Vivo Translation
Source: Adv Healthc Mater. 2026 Aug 26;15(36):e71646. doi: 10.1002/adhm.71646 (PMC13612937; doi:10.1002/adhm.71646)
Supplement: Supplementary file 1 [file ADHM-15-0-s001.docx]

**Supporting Information**

**Plasma Gelation as an Overlooked Determinant of Transfection Efficiency in Protein Corona-Coated Lipid Nanoparticles: Implications for In Vitro-In Vivo Translation**

*Serena Renzi^1#^, Luca Digiacomo^2#^, Francesca Giulimondi^1^, Daniela Pozzi^1,5^, Junbiao Wang^3^, Augusto Amici^3^, Cristina Marchini^3*^, Chiara Cassone^1^, Alessandra Zingoni^1^, Fabrizio Marra^4,5^, Luca Buccini^6^, Francesco Mura^5^, Valentina De Lorenzi^7^, Francesco Cardarelli^7^, Giulio Caracciolo^1,5*^*

*^1^Department of Molecular Medicine, Sapienza University of Rome, 00161 Rome, Italy*

*^2^Department of Medico-Surgical Sciences and Biotechnologies, Sapienza University of Rome, Corso della Repubblica 79, Latina, 04100, Italy*

*^3^School of Biosciences and Veterinary Medicine, University of Camerino, 62032 Camerino, Italy*

*^4^Department of Astronautics, Electrical and Energy Engineering Sapienza University of Rome, Rome, Italy*

*^5^Research Center on Nanotechnologies applied to Engineering (CNIS) Sapienza University of Rome, Rome, Italy*

*^6^Department of Basic and Applied Sciences for Engineering (SBAI), Sapienza University of Rome, Rome, Italy*

*^7^Laboratorio NEST, Scuola Normale Superiore, 56127 Pisa, Italy*

^#^S. Renzi and L. Digiacomo contributed equally to this work

^*^Corresponding author. Email: [cristina.marchini@unicam.it](mailto:cristina.marchini@unicam.it); [giulio.caracciolo@uniroma1.it](mailto:giulio.caracciolo@uniroma1.it)

**Figure S1**. **Representative Transmission Electron Microscopy (TEM) images for formulations F1-F5**. Multiple fields are shown for each formulation to illustrate particle structure and morphology. Nanoparticles (NPs) F1-F4 predominantly display rounded multilamellar vesicular structures, whereas F5 appears less well-defined and more heterogeneous, with smaller, poorly contrasted aggregates. Scale bars are reported within the individual panels.

**Figure S2**. **Size distributions and zeta potential distributions of formulations F1-F5 in buffer and in human plasma (HP)**. Size distributions for (a) F1, (b) F2, (c) F3, (d) F4, and (e) F5. Zeta potential distributions for (f) F1, (g) F2, (h) F3, (i) F4, and (j) F5.

**Figure S3**. **Calcium-Dependent Gel Network Formation Revealed by Structural and Rheological Characterization.** (a) Scanning Electron Microscopy (SEM) characterization of the gelled component formed in standard medium upon cell treatment with F1+HP. SEM images revealed a heterogeneous interconnected matrix characterized by elongated structural features and irregular interstitial spaces. Although the dehydration process required for SEM preparation may induce structural rearrangements or partial collapse of the soft gel network, the images reveal a hierarchical organization of the dried material. Increasing magnification showed a continuous rough surface with locally aligned domains, consistent with a highly organized dried microstructure. The corresponding sample in calcium-free medium remained completely liquid, with no visible phase separation; therefore, SEM analysis was not applicable. CNIS denotes the Center for Nanotechnology Applied to Engineering of Sapienza University of Rome where SEM experiments were performed (b) Rheological characterization of the standard culture medium (control) and the F1 formulation exposed to HP, both in standard and calcium-free media. The visibly gelled component embedded in the standard medium was characterized. The control curve (standard medium, dotted line) exhibits the typical behavior of a Newtonian fluid, with nearly constant viscosity across the entire range of shear rates investigated. A slight increase in apparent viscosity at low shear rates was observed for the sample in calcium-free medium (dash-dot line) suggesting weak interparticle or protein-nanoparticle interactions that modestly hinder flow under quiescent conditions. In contrast, the whole F1-HP mixture in standard medium (solid line) shows a pronounced increase in viscosity and a clear shear-thinning behavior, consistent with the formation of a percolating or crosslinked network mediated by calcium ions. This behavior indicates a transition toward a non-Newtonian, pseudoplastic flow at low shear rates, characteristic of soft gel systems.

**Protein corona composition in different cell media**

To assess the potential effect of calcium on protein corona composition, we performed one-dimensional SDS-PAGE analysis of F1 incubated with HP under different biologically relevant conditions. Specifically, water was used as a control, while standard medium and calcium-free medium were employed to mimic the extracellular environments of interest. In addition, calcium-free medium supplemented with CaCl₂ to a final concentration of 1.2 mM (physiological concentration) was included to evaluate the specific contribution of calcium. The results are shown in Figure S4. For each condition, both HP-only ("blank") samples and F1+HP samples were loaded in duplicate (Figure S4a), and the corresponding densitometric profiles were calculated. Blank-subtracted intensity profiles for each F1+HP sample are reported in Figure S4 b-f. The higher abundance of corona-associated proteins observed in water, as evidenced by the clearly more intense bands in Figure S4a (lanes 6 and 15), likely reflects the lower perturbation of the preformed F1-HP complexes. In contrast, transfer to standard DMEM, calcium-free DMEM, or calcium-free DMEM supplemented with CaCl₂ exposes the complexes to higher ionic strength and a chemically more complex environment, which can screen electrostatic protein-lipid interactions, alter interfacial hydration, and promote selective protein desorption and corona rearrangement, thereby reducing the overall protein loading at equilibrium. This interpretation is consistent with previous evidence showing that ionic strength modulates protein-surface interactions by screening electrostatic forces and thereby altering protein adsorption-desorption equilibria and corona organization (Cantarutti, Cristina, et al. "The corona of protein-gold nanoparticle systems: the role of ionic strength." Physical Chemistry Chemical Physics 24.3 (2022): 1630-1637.).

The integrated band intensities are summarized in Figure S4 g, while the heatmap of pairwise correlation coefficients shown in Figure S4 h provides a quantitative measure of the similarity between the protein corona profiles obtained under the different experimental conditions. Overall, the results indicate that the patterns in water displayed a markedly higher total intensity and greater compositional complexity than those formed under the other conditions. In particular, the corresponding densitometric profile is characterized by a dominant band at approximately 80 kDa, accompanied by a series of peaks in the 55-75 kDa range and additional contributions at both lower and higher molecular weights. Conversely, the corona profiles obtained in cell culture media were more similar to one another. The profile in standard medium is distinguished by a prominent band at approximately 60 kDa, and further peaks at low molecular weights (10-15 kDa), at 20-30 kDa, 40 kDa and 80 kDA . Finally, the protein patterns formed in calcium-free medium and calcium-free medium supplemented with CaCl₂ exhibited highly similar densitometric profiles, comparable total intensities, and the highest pairwise correlation coefficient (0.723). Only minor differences were observed between these conditions, including the appearance of a secondary peak at approximately 25 kDa in the CaCl₂-supplemented medium. Taken together, these data indicate that the protein patterns depend on the environment in which the systems are embedded, but calcium does not induce major changes in the global profile.

**Figure S4. Modulation of the F1 Protein Corona by the Dispersion Medium.** (a) Gel image showing HP and F1+HP samples prepared in water, standard medium, calcium-free medium, and calcium-free medium supplemented with 1.2 mM CaCl₂. (b-e) Corresponding one-dimensional intensity profiles of the F1+HP samples after subtraction of the HP signal. (f) Total band intensity. (g) Correlation heatmap of the intensity profiles. Statistical analysis was performed by one-way ANOVA followed by Tuckey’s post hoc test. Statistical significance is indicated as follows: *p < 0.05, **p < 0.01. Comparisons with p > 0.05 are not shown.

**Figure S5**. **Transfection efficiency (TE) and cell viability of bare F1 in standard and calcium-free citrated media.** HEK-293 cells were treated with bare F1 in media supplemented with or without sodium citrate (12.5 mM) for 3 h, then (a) TE and (b) cell viability were measured after 48h. Statistical analysis was performed by two-way ANOVA followed by Šidák’s post hoc test. Statistical significance is indicated as follows: **** p < 0.0001; * p < 0.05; comparisons with p > 0.05 are not shown.

**Figure S6. Colloidal stability of F1+HP in standard cell culture medium supplemented with sodium citrate.** (a) hydrodynamic size and (b) polydispersity index (PdI) of F1+HP resuspended in standard medium containing increasing concentrations of sodium citrate. The shaded bands represent the mean ± standard deviation of F1+HP dispersed in water. Measurements were performed immediately after NP resuspension (t = 0) and after 3 h of incubation. Sodium citrate did not affect NP size and only marginally influenced PdI, with no statistically significant differences detected (two-way ANOVA followed by Šidák's post hoc test). Overall, the colloidal stability of the NPs was maintained upon exposure to standard medium supplemented with sodium citrate.

**Figure S7**. **Effect of Sodium Citrate Concentration on the Protein Corona Profile of F1.** (a) SDS-PAGE gel image of F1+HP samples incubated in standard medium supplemented with different concentrations of sodium citrate. The control sample corresponds to F1+HP incubated in water supplemented with the highest sodium citrate concentration (12.5 mM). (b) Corresponding lane intensity profiles, (c) total lane intensities, (d) heatmap of pairwise correlation coefficients. The results show that both the amount and composition of the protein corona formed on F1 following exposure to HP differ markedly depending on the surrounding medium, with substantial differences observed between samples dispersed in water and those dispersed in sodium citrate-supplemented standard medium, irrespective of the sodium citrate concentration. In contrast, the protein patterns, densitometric profiles, and total lane intensities obtained in sodium citrate-supplemented standard medium are similar across all the investigated citrate concentrations, as confirmed by the high pairwise correlation coefficients. Although some citrate-dependent variations in the intensity of selected bands can be observed, particularly at approximately 27 and 64 kDa, the data indicates that the surrounding microenvironment exerts a much greater influence on protein corona composition than sodium citrate concentration itself. Statistical analysis was performed by one-way ANOVA followed by Tuckey’s post hoc test. Statistical significance is indicated as follows: **** p < 0.0001; comparisons with p > 0.05 are not shown.

**Figure S8**. **Transfection efficiency of F1 in buffer and after exposure to HP from human donors, and corresponding HEK-293 cell viability**. TE for each donor in (a) standard medium, and (b) calcium-free medium. TE distribution for all the donors in (c) standard medium and (d) calcium-free medium. Cell viability upon treatment of F1 exposed to plasma from each donor in (e) standard medium, and (f) calcium-free medium. Cell viability distribution for all the donors in (g) standard medium and (h) calcium-free medium. Statistical analysis was performed by one-way ANOVA followed by Dunnett’s post hoc test for individual data (Panels a, b, e, f), and by Student’s t-test for grouped data (two-class comparisons, Panels c, d, g, h). Statistical significance is indicated as follows: n.s. = not significant; *p < 0.05, **p < 0.01, ***p < 0.001, ****p < 0.0001.

**Figure S9**. **Transfection efficiency of F1 to HP in heparinized vials, and corresponding HEK-293 cell viability.** HEK-293 cells were treated in standard DMEM using coronated F1 with increasing amount of heparin (concentration ranging from 2 U/mL to 16 U/mL). TE was represented by orange dots whereas cell viability by green dots. Data are presented as mean ± SD (N=4). No gel formation was observed at heparin concentrations ≥ 4U/mL but just at the lowest (i.e. 2 U/mL, gel clearly apparent by visual inspection). Heparin itself exerted a non-negligible and deleterious effect on transfection efficiency, making it unsuitable for studying the relationship between PC formation and gene delivery system performance. The absence of gelation in heparinized plasma, despite the presence of free Ca²⁺, indicates that the phenomenon observed in citrated or EDTA plasma is not due only to simple physicochemical interactions between ions and proteins but involves activation of the coagulation cascade.

**Figure S10**. **Flow cytometry experiments of HEK-293 cells treated with F1 in buffer, or in HP, both in standard and calcium-free medium.**  Contour plots corresponding to (a) bare F1 in standard medium, (b) bare F1 in calcium-free medium, (c) HP-exposed F1 in standard medium, and (d) HP-exposed F1 in calcium-free medium. Data are exported from Flow Jo, as contour plots. Axis refer to PE-A and SSC_H signals.

**Figure S11. Quantitative colocalization analysis of fluorescence confocal images** between (a-c) lipid and DNA channels, (d-f) lipid and lysosome channels, and (g-i) DNA and lysosome channels, expressed as Pearson’s and Manders’ coefficients. Colocalization analysis was performed in ImageJ after thresholding according to the Costes method. No significant trends or differences were observed among the tested conditions.

The raw data underlying **Figures 4-6**, including time-lapse fluorescence image sequences used for Raster Image Correlation Spectroscopy (RICS), static multi-channel acquisition used for colocalization analysis, and image-derived Mean Square Displacement (iMSD) analyses, have been deposited on Zenodo and are publicly available at the following DOI: https://doi.org/10.5281/zenodo.18763832. These datasets support the findings presented in **Figure 4**, which demonstrates reduced nanoparticle diffusion in the extracellular space under plasma gelation conditions, and in **Figure 6**, which shows that intracellular nanoparticle mobility remains unaffected by either plasma exposure or gelation, as determined through quantitative iMSD analysis in HEK-293 cells.
